# Left Ventricular Ejection Fraction Predicts Outcomes in Different Subgroups of Patients Undergoing Coronary Angiography

**DOI:** 10.3390/jcm14155219

**Published:** 2025-07-23

**Authors:** Henning Johann Steffen, Tobias Schupp, Mohammad Abumayyaleh, Lasse Kuhn, Philipp Steinke, Jonas Dudda, Kathrin Weidner, Jonas Rusnak, Mahboubeh Jannesari, Fabian Siegel, Daniel Duerschmied, Michael Behnes, Ibrahim Akin

**Affiliations:** 1 Cardiology, Haemostasis, and Medical Intensive Care, Medical Faculty Mannheim, University Medical Centre Mannheim, Heidelberg University, 68167 Mannheim, Germany; henningjohann.steffen@umm.de (H.J.S.); mohammad.abumayyaleh@umm.de (M.A.); lasse.kuhn@stud.uni-heidelberg.de (L.K.); philipp.steinke@stud.uni-heidelberg.de (P.S.); jonas.dudda@umm.de (J.D.); kathrin.weidner@umm.de (K.W.); daniel.duerschmied@umm.de (D.D.); michael.behnes@umm.de (M.B.); ibrahim.akin@umm.de (I.A.); 2 European Center for AngioScience (ECAS), German Centre for Cardiovascular Research (DZHK), Partner Site Heidelberg/Mannheim, and Centre for Cardiovascular Acute Medicine Mannheim (ZKAM), Medical Centre Mannheim and Medical Faculty Mannheim, Heidelberg University, 68167 Mannheim, Germany; 3 Department of Cardiology, Angiology and Pneumology, University Hospital Heidelberg, 69120 Heidelberg, Germany; jonas.rusnak@med.uni-heidelberg.de; 4 Department of Biomedical Informatics, Center for Preventive Medicine and Digital Health (CPD), Medical Faculty Mannheim, Heidelberg University, 68167 Mannheim, Germany; mahboubeh.jannesari@medma.uni-heidelberg.de (M.J.); fabian.siegel@medma.uni-heidelberg.de (F.S.); 5 Helmholtz-Institute for Translational AngioCardioScience (HI-TAC), Max Delbrück Center for Molecular Medicine, Helmholtz Association (MDC), Heidelberg University, 10115 Berlin, Germany

**Keywords:** LVEF, coronary angiography, heart failure, acute myocardial infarction, long-term outcomes, risk stratification

## Abstract

**Objectives:** To evaluate the long-term prognostic value of left ventricular ejection fraction (LVEF) in consecutive patients undergoing invasive coronary angiography (CA). **Background:** LVEF is a key prognostic marker in cardiovascular disease, but its value across different clinical indications for CA remains insufficiently characterized. **Methods:** Consecutive patients undergoing CA between January 2016 and August 2022 were retrospectively included at one institution. Patients were stratified into four LVEF groups: ≥ 55%, 45–54%, 35–44%, and <35%. The primary endpoint was rehospitalization for heart failure (HF) at 36 months. Secondary endpoints were acute myocardial infarction (AMI) and coronary revascularization. Kaplan–Meier and multivariable Cox regression analyses were conducted within the entire study cohort and pre-defined subgroups. **Results:** A total of 6888 patients were included (median age: 71 years; 65.2% males). LVEF < 35% was associated with a higher comorbidity burden and more extensive coronary artery disease (e.g., three-vessel CAD: 38.6% vs. 20.7%, *p* < 0.001). Event rates for HF rehospitalization and AMI increased progressively with declining LVEF, while revascularization rates varied across categories. Statistically significant differences across LVEF groups were observed for all three endpoints in unadjusted analyses (log-rank *p* < 0.001). In multivariable models, LVEF < 35% independently predicted HF rehospitalization (HR = 3.731, *p* < 0.001) and AMI (HR = 4.184, *p* < 0.001), but not revascularization (HR = 0.867, *p* = 0.378). The prognostic association was demonstrated across all subgroups stratified by age, sex, subtype of acute coronary syndrome, and CAD severity. **Conclusions:** Reduced LVEF is an independent predictor of HF rehospitalization and AMI in patients undergoing coronary angiography, irrespective of its indication, whereas no independent association was observed with coronary revascularization.

## 1. Introduction

Coronary artery disease (CAD), the most prevalent form of cardiovascular disease (CVD), affects more than 126 million individuals worldwide and is a leading contributor to the more than 9 million annual deaths attributed to CVD [1,2]. Invasive coronary angiography (CA) remains the cornerstone diagnostic tool for assessing CAD severity and treatment, particularly in patients with acute coronary syndromes or unexplained symptoms [3]. However, the long-term prognostic determinants in consecutive patients undergoing CA remain unclear, as previous studies predominantly investigated outcomes in pre-selected subgroups, such as acute myocardial infarction (AMI) or heart failure (HF) [4].

In patients undergoing CA, left ventricular ejection fraction (LVEF) is a key parameter for both clinical decision-making and risk stratification. As a measure of systolic function, LVEF indicates therapy in patients with HF with reduced ejection fraction (HFrEF) and is strongly associated with mortality, HF progression, and adverse cardiovascular events [5,6,7]. However, the prognostic implications of LVEF categories across a wider clinical spectrum, especially in patients without acute HF at presentation, remain unclear. In particular, the risk associated with moderately reduced (35–45%) and preserved (>45%) LVEF has not been fully explored in real-world angiographic settings. Growing evidence challenges the binary classification of LVEF, suggesting a continuum of risk across LVEF strata [8,9]. Even patients with mildly reduced LVEF may face increased risks of HF rehospitalization and major adverse events. This paradigm shift is clinically relevant, as HF with preserved ejection fraction (HFpEF) now constitutes the majority of HF presentations, despite the lack of disease-modifying therapies [8,10].

Therefore, this study investigates the association between LVEF strata and the risk of HF-related rehospitalization, AMI, and coronary revascularization at 36 months in consecutive patients undergoing CA. Subgroup analyses were performed, stratified by age, sex, and clinical indications for CA, as well as the extent of CAD.

## 2. Methods

### 2.1. Study Population, Design, and Data Collection

This retrospective observational study included all consecutive patients of at least 18 years of age who underwent invasive CA at the University Medical Centre Mannheim (UMM), Germany, between January 2016 and August 2022. Patients were identified using German Operation and Procedure Classification System (OPS) codes. Relevant clinical data were retrieved from the institutional electronic hospital information system (SAP^®^, Walldorf, Germany), including demographic characteristics, cardiovascular risk factors, comorbidities, clinical presentation, angiographic findings, and discharge medications. Patients who underwent multiple CA procedures during the study period were only included once. This study was approved by the Ethics Committee II of the Medical Faculty Mannheim, Heidelberg University (reference: 2022-829), and conducted in accordance with the principles of the Declaration of Helsinki. The registry was registered with the German Clinical Trials Register (DRKS00034765).

All CA procedures were performed by board-certified interventional cardiologists during routine clinical practice in accordance with the current guidelines of the European Society of Cardiology (ESC) and the European Association for Cardio-Thoracic Surgery (EACTS) on myocardial revascularization [3].

### 2.2. Inclusion and Exclusion Criteria

All patients aged ≥18 years undergoing invasive CA during the study period were eligible. To ensure a reliable LVEF-based subgroup analysis, only patients with documented LVEF at the baseline were included. LVEF was assessed during index hospitalization, primarily via transthoracic echocardiography; if unavailable, values from left ventriculography were used. Patients without documented LVEF during index hospitalization were excluded. No additional exclusion criteria based on specific comorbidities (e.g., recent acute coronary syndrome or renal dysfunction) were applied. Potential confounding from such conditions was addressed through multivariable adjustment, as detailed in the Section 2.5.

### 2.3. LVEF Stratification

Patients were stratified into four groups based on LVEF measured at index hospitalization in accordance with previously published studies using comparable cut-offs [11,12,13]: ≥55% (normal/preserved systolic function), 45–54% (mild systolic dysfunction), 35–44% (moderate systolic dysfunction) and <35% (severe systolic dysfunction). LVEF measurements followed established guidelines from the American Society of Echocardiography (ASE) and the European Association of Cardiovascular Imaging (EACVI) [14].

### 2.4. Study Endpoints

The primary endpoint was rehospitalization for HF within 36 months of the follow-up. Secondary endpoints included acute myocardial infarction (AMI) at 36 months and coronary revascularization at 36 months. All clinical endpoints were identified based on International Classification of Diseases (ICD-10) and OPS codes from discharge summaries, ensuring standardized and validated endpoint classification.

### 2.5. Statistical Methods

Continuous variables are expressed as the median and interquartile range (IQR) or the mean ± standard deviation (SD), as appropriate. Group comparisons were performed using the Kruskal–Wallis test or one-way ANOVA for continuous variables, and the Chi-square test or Fisher’s exact test for categorical variables. Time-to-event data for HF-related rehospitalization, AMI, and coronary revascularization were analyzed using Kaplan–Meier curves, with group comparisons made using the log-rank test. Cox proportional hazard models were used to evaluate the association between LVEF categories and clinical endpoints.

Multivariable models were adjusted for relevant baseline covariates, including age, sex, diabetes mellitus, prior CAD, MI, prior coronary artery bypass grafting (CABG), chronic kidney disease (CKD), type of acute coronary syndrome (STEMI (ST-elevation myocardial infarction) or NSTEMI (non-ST-elevation myocardial infarction)), atrial fibrillation (AF), decompensated HF at index hospitalization, and the use of evidence-based pharmacotherapies at discharge, including ACE (angiotensin-converting enzyme) inhibitors or ARBs (angiotensin II receptor blockers), beta-blockers, aldosterone antagonists, and SGLT2 (sodium–glucose cotransporter 2) inhibitors. 

All covariates were assessed for missing values. Patients with incomplete data for any of the included variables were excluded from the regression analyses via listwise deletion (complete case analysis). No imputation was performed. Of the 7691 patients initially identified, 803 were excluded due to missing LVEF and a further 308 (4.0%) due to missing values in one or more covariates, resulting in a final cohort of 6581 patients included in the multivariable analyses.

Subgroup analyses were stratified by age (<70 vs. ≥70 years), sex, extent of CAD, and ACS subtype with regard to the primary endpoint. Hazard ratios (HRs) with 95% confidence intervals (CIs) are reported. Statistical significance was defined as a two-sided *p*-value ≤ 0.05. Statistical analyses were performed using IBM SPSS Statistics (Version 25, IBM Corp., Armonk, NY, USA).

## 3. Results

### 3.1. Study Population

**Figure 1:**
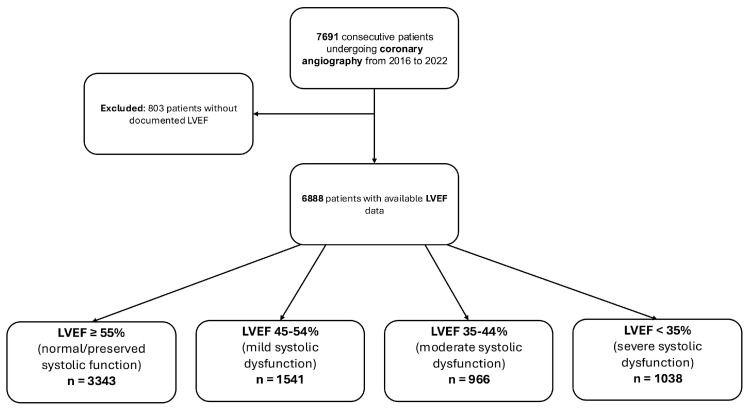
Study flowchart illustrating the inclusion and stratification of patients according to baseline left ventricular ejection fraction (LVEF).

Between January 2016 and August 2022, a total of 7691 patients underwent invasive CA at the University Medical Centre Mannheim. After excluding 803 patients with missing LVEF data during index hospitalization, 6888 patients were included in the final analysis. The median age was 71 years and 65.2% of the patients were males. Patients were stratified into four LVEF groups: LVEF ≥ 55% (*n* = 3343; 48.5%), LVEF of 45–54% (*n* = 1541; 22.4%), LVEF of 35–44% (*n* = 966; 14.0%), and LVEF < 35% (*n* = 1038; 15.1%). Baseline characteristics differed significantly across the LVEF strata (Table 1). Median age differed significantly across groups, with patients in the LVEF ≥ 55% group being younger (median of 68 years) compared to those with LVEF of 45–54% (median of 71 years), 35–44% (median of 72 years), and <35% (median of 70 years; *p* = 0.001). The proportion of males increased with decreasing LVEF (i.e., LVEF ≥ 55%: 58.7%; LVEF of 45–54%: 69.0%, LVEF of 35–44%: 69.8%; and LVEF < 35%: 75.6%; *p* = 0.001). The prevalence of diabetes mellitus (i.e., LVEF ≥ 55%: 23.7%; LVEF of 45–54%: 27.4%; LVEF of 35–44%: 29.7%; and LVEF < 35%: 26.8%; *p* = 0.001) and CKD (i.e., LVEF ≥ 55%: 4.2%; LVEF of 45–54%: 5.6%; LVEF of 35–44%: 6.8%; and LVEF < 35%: 8.5%; *p* = 0.001) increased with decreasing LVEF. A similar trend was observed regarding the rates of congestive HF (i.e., LVEF ≥ 55%: 5.0%; LVEF of 45–54%: 10.8%; LVEF of 35–44%: 13.8%; and LVEF < 35%: 20.0%; *p* = 0.001). Notably, acute decompensated HF on admission was more frequently observed in patients with lower LVEF (i.e., LVEF ≥ 55%: 6.2%; LVEF of 45–54%: 10.0%; LVEF of 35–44%: 21.7%; and LVEF < 35%: 69.5%; *p* = 0.001) (Table 1 and Figure 1).

The severity and extent of CAD increased progressively with decreasing LVEF. The prevalence of three-vessel disease was highest in patients with reduced LVEF (i.e., LVEF ≥ 55%: 20.7%; LVEF of 45–54%: 33.5%; LVEF of 35–44%: 39.3%; and LVEF < 35%: 38.6%; *p* = 0.001), and patients with lower LVEF were more frequently sent to CABG (LVEF ≥ 55%: 3.6%; LVEF of 45–54%: 4.7%; LVEF of 35–44%: 7.1%; and LVEF < 35%: 5.1%; *p* = 0.001). Left main coronary artery involvement and coronary chronic total occlusions were also more frequently observed in patients with reduced LVEF. Correspondingly, PCI rates varied across the groups (i.e., LVEF ≥ 55%: 36.6%; LVEF of 45–54%: 52.0%; LVEF of 35–44%: 48.0%; and LVEF < 35%: 42.6%; *p* = 0.001). Furthermore, in-hospital all-cause mortality increased markedly with decreasing LVEF (LVEF ≥ 55%: 0.9%; LVEF of 45–54%: 2.5%; LVEF of 35–44%: 4.8%; and LVEF < 35%: 16.6%; *p* = 0.001) (Table 2).

### 3.2. Prognostic Impact of LVEF

**Figure 2:**
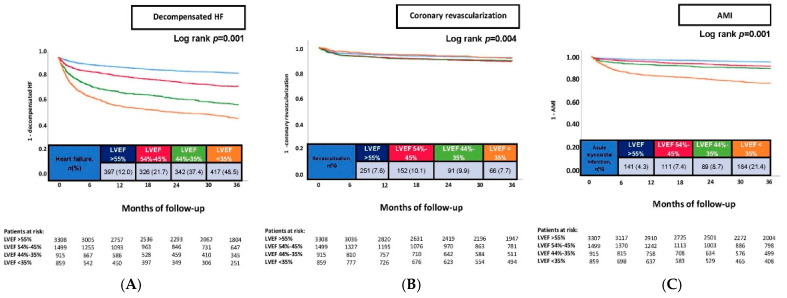
Prognostic impact of left ventricular ejection fraction (LVEF) in consecutive patients on the risk of HF-related rehospitalization at 36 months (**A**), coronary revascularization at 36 months (**B**), and AMI at 36 months (**C**). AMI, acute myocardial infarction; HF, heart failure; and LVEF, left ventricular ejection fraction.

At 36 months, the primary endpoint of HF-related rehospitalization occurred significantly more often in patients with lower LVEF values (LVEF ≥ 55%: 12.0%; LVEF of 45–54%: 21.7%; LVEF of 35–44%: 37.4%; and LVEF < 35%: 48.5%; *p* = 0.001) (Table 2, Figure 2A). A similar pattern was observed for the secondary endpoint of AMI (LVEF ≥ 55%: 4.3%; LVEF of 45–54%: 7.4%; LVEF of 35–44%: 8.7%; and LVEF < 35%: 21.4%; *p* = 0.001) (Table 2, Figure 2C). In contrast, the frequency of coronary revascularization was highest in patients with LVEF of 45–54% (LVEF ≥ 55%: 7.6%; LVEF of 45–54%: 10.1%; LVEF of 35–44%: 9.9%; and LVEF < 35%: 7.7%; *p* = 0.008) (Table 2, Figure 2B).

### 3.3. Multivariable Cox Regression Analyses

After multivariable adjustment, lower LVEF remained an independent predictor of adverse clinical outcomes at 36 months (Table 3). Compared to patients with preserved LVEF (≥55%), the risk of HF-related rehospitalization increased progressively across LVEF strata (LVEF of 45–54%: HR = 1.826; 95% CI: 1.573–2.121; *p* = 0.001; LVEF of 35–44%: HR = 2.948; 95% CI: 2.523–3.446; *p* = 0.001; and LVEF < 35%: HR = 3.731; 95% CI: 3.168–4.394; *p* = 0.001). Beyond LVEF, several clinical covariates were independently associated with increased risk of HF-related rehospitalization, including prior CAD (HR = 1.631; *p* = 0.001), decompensated HF at admission (HR = 1.607; *p* = 0.001), AF (HR = 1.223; *p* = 0.004), and diabetes mellitus (HR = 1.151; *p* = 0.044).

Similarly, lower LVEF values were associated with a higher risk of AMI (LVEF of 45–54%: HR = 1.667; *p* = 0.001; LVEF of 35–44%: HR = 1.988; *p* = 0.001; and LVEF < 35%: HR = 4.184; *p* = 0.001) whereas no statistically significant association was observed between LVEF and the risk of coronary revascularization (LVEF of 45–54%: HR = 1.172; *p* = 0.136; LVEF of 35–44%: HR = 1.084; *p* = 0.543; and LVEF < 35%: HR = 0.867; *p* = 0.378) compared to patients with LVEF ≥ 55%.

### 3.4. Subgroup Analyses

Subgroup analyses demonstrated that the association between reduced LVEF and HF-related rehospitalization remained consistent across clinically relevant strata, with especially pronounced effects in younger patients and those with acute coronary syndromes (Table 4). Stratified analyses by age, sex, ACS subtype, and CAD extent confirmed the prognostic significance of reduced LVEF across all clinical settings. The effect was particularly pronounced in younger patients; those aged < 70 years with LVEF < 35% had a more than three-fold increased risk (HR = 3.874; 95% CI: 2.783–5.393), compared to an HR of 2.338 in those aged ≥ 70 years (95% CI: 1.793–3.050). Both sexes exhibited significant associations, with HRs of 3.363 (95% CI: 2.459–4.599) in males and 3.061 (95% CI: 2.048–4.579) in females. The impact of reduced LVEF was also evident across ACS presentations, including NSTEMI (HR = 3.420; 95% CI: 2.349–4.979), STEMI (HR = 4.028; 95% CI: 2.351–6.892), and unstable angina (HR = 4.214; 95% CI: 2.800–6.340) (*p* = 0.001 for all comparisons).

## 4. Discussion

This study provides robust evidence that LVEF is a strong and independent predictor of adverse cardiovascular outcomes, including HF rehospitalization and AMI in patients undergoing CA during a 36-month follow-up period. Importantly, we demonstrated a continuous relationship between decreasing LVEF and increasing event rate, with patients in the lowest LVEF category (<35%) experiencing the highest risk of adverse outcomes. These findings extend the prognostic relevance of LVEF beyond traditional HF cohorts in a large, consecutive population undergoing invasive CA. Adverse outcomes in patients with impaired LVEF were demonstrated across all analyzed subgroups, suggesting an independent association of HF with adverse long-term prognosis, irrespective of the indication of invasive CA.

The measurement of LVEF represents a well-established cornerstone for risk stratification in patients with HF and AMI [5]. However, most of the existing literature evaluates highly selected populations, often excluding patients with preserved or mildly reduced ejection fraction [8,9]. In contrast, our study included an all-comer cohort undergoing CA, which enabled a more representative and comprehensive understanding of the prognostic role of LVEF in real-world clinical settings. Our findings support the paradigm that LVEF should not be viewed through a binary lens (i.e., reduced vs. preserved), but rather as a continuous variable that reflects gradations of cardiovascular risk, demonstrating increased risk of HF-related rehospitalization with decreasing LVEF across all analyzed subgroups [5,15].

Notably, the association between reduced LVEF and adverse outcomes was particularly pronounced in patients aged < 70 years. This aligns with previous observations from our group [16], where younger patients exhibited a steeper risk trajectory, despite a lower absolute burden of comorbidities. One possible explanation is the greater hemodynamic and structural myocardial response to ischemia in younger patients, which may exacerbate systolic dysfunction over time, if not addressed early. Sex-specific analyses showed similar trends, although male patients were over-represented in the reduced LVEF strata [17]. These differences likely reflect underlying disparities in coronary disease burden and ventricular remodeling; however, further sex-disaggregated analyses are needed to clarify whether treatment effects differ across LVEF categories in men and women.

Our data demonstrated a correlation between lower LVEF and more advanced CAD, including a higher prevalence of three-vessel CAD and chronic total occlusions. This supports the notion that the cumulative ischemic burden contributes to progressive left ventricular dysfunction and adverse remodeling [18,19]. Conversely, patients with preserved LVEF were more likely to have non-obstructive or normal coronary arteries, further underscoring the interaction between anatomical disease and functional impairment [20]. Interestingly, patients with reduced LVEF were less likely to undergo complete revascularization or PCI, despite having more extensive CAD. This may reflect perceived procedural risk, reduced myocardial viability, or a preference for conservative therapy. However, in our multivariable models, LVEF was not independently associated with revascularization, indicating that other factors—such as anatomical disease burden—likely influenced revascularization decisions. Emerging data suggest that incomplete revascularization may contribute to adverse outcomes in these patients and warrants further prospective study [21,22].

Our findings have substantial therapeutic implications. Current European and American guidelines emphasize the role of LVEF in selecting patients for therapies such as beta-blockers, mineralocorticoid receptor antagonists, angiotensin receptor neprilysin inhibitors (ARNis), and implantable cardioverter-defibrillators [5]. However, most recommendations focus on patients with LVEF < 40%, leaving an evidence gap for patients within the 40–55% range. Importantly, the observed stepwise risk increase across adjacent LVEF groups supports clinical vigilance not only in patients with severely reduced LVEF, but also in those with mid-range or mildly reduced values, who have traditionally received less aggressive follow-ups or therapies. Recent randomized trials, such as EMPEROR-Preserved and DELIVER, have shown that sodium–glucose cotransporter 2 (SGLT2) inhibitors reduce HF hospitalization in patients with LVEF > 40% [23,24]. While these data have led to updates in guideline recommendations, their implementation in routine practice remains inconsistent. Our findings support the broader application of risk-guided therapy, independent of categorical LVEF cutoffs, to reduce adverse cardiovascular outcomes in intermediate-risk patients. Furthermore, while treatment with evidence-based therapies such as beta-blockers, SGLT2 inhibitors, and RAAS (Renin–Angiotensin–Aldosterone System) blockade was more common in patients with reduced LVEF, our multivariable models adjusted for these variables to mitigate potential confounding. Thus, the prognostic associations of LVEF were independent of discharge pharmacotherapy. Nonetheless, residual confounding related to unmeasured treatment factors (e.g., dosing, medication adherence, or the specific use of ARNis) cannot be fully excluded.

This is especially relevant given the increasing burden of HFpEF, which now accounts for more than 50% of all HF cases and is associated with significant morbidity, hospitalization, and reduced quality of life despite a relatively preserved LVEF [8,10]. Therefore, earlier identification of at-risk patients using echocardiographic and angiographic data, including borderline LVEF values, may offer an opportunity for earlier intervention and more effective disease modification. Our findings are consistent with the 2023 ESC guidelines for acute coronary syndromes, which emphasize biomarker- and LVEF-guided risk stratification in addition to early invasive strategies for high-risk patients following PCI [25]. Furthermore, recent data from the PRAISE Registry [26] on prognoses in ACS patients support our observed gradient of risk across LVEF categories, underscoring the broader applicability of our findings.

From a clinical perspective, our results support the routine assessment and interpretation of LVEF in all patients undergoing coronary angiography, not only for therapeutic decision-making but also for long-term risk stratification based on its continuous association with outcome risk even across narrow functional differences. This is particularly relevant in the context of aging populations and the rising prevalence of multimorbidity, in which traditional risk markers may be insufficiently sensitive. Beyond its diagnostic role, LVEF can aid in determining the intensity and frequency of post-discharge monitoring, guide referrals for cardiac rehabilitation, and provide patient education regarding symptoms and lifestyle modifications. The integration of LVEF with additional biomarkers, such as N-terminal pro–B-type natriuretic peptide (NT-proBNP), coronary flow reserve, and myocardial strain imaging, may further enhance its predictive value and merits future investigation. Together, these findings highlight the central role of LVEF in guiding both clinical decision-making and long-term monitoring strategies for patients undergoing coronary angiography.

## 5. Limitations

This retrospective study was subject to residual confounding from unmeasured variables, such as frailty, medication adherence, and socioeconomic status. LVEF was assessed at the baseline, primarily via transthoracic echocardiography, following guideline standards, although variability in technique may have affected classification. The follow-up relied on electronic records and diagnostic coding, which may be prone to misclassification. We did not perform formal multicollinearity testing, such as variance inflation factor analysis, which could further validate our multivariable model. This represents an area for future methodological refinement. Serial LVEF measurements and treatment adjustments during the follow-up period were unavailable. Additionally, the exclusion of patients with missing LVEF (803 patients) or missing data in any of the covariates used in the regression models (308 patients) may introduce a degree of selection bias. However, the overall rate of complete cases was high (85.6%), and the proportion of missingness per variable was low (<15%). No imputation was performed due to the observational nature of the study and the robustness of the sample size. Potential selection bias cannot be excluded given the observational nature of our study. Additionally, although we adjusted for major classes of discharge medication (beta-blockers, ACEi/ARB, aldosterone antagonists, and SGLT2 inhibitors) in our multivariable models, the possibility of residual confounding due to unmeasured differences in treatment intensity (e.g., drug dosages, ARNI use, and adherence) remains. Furthermore, no adjustment for cardiac troponin I levels was performed according to its variable measurement during index hospitalization.

## 6. Conclusions

Reduced LVEF was independently associated with a higher risk of HF rehospitalization and AMI at 36 months across all subgroups of patients undergoing invasive coronary angiography. This relationship followed a stepwise gradient across LVEF categories, including mid-range and preserved ejection fractions. No independent association was found between LVEF and coronary revascularization in adjusted analyses. These findings support the integration of LVEF into prognostic models for all patients undergoing CA irrespective of clinical presentation. Stratification by LVEF may identify higher-risk individuals beyond those with guideline-defined HFrEF, and highlight patients who may benefit from intensified follow-ups and the earlier initiation of risk-modifying strategies.

## Figures and Tables

**Table 1:** Baseline characteristics.

	LVEF ≥ 55% (*n* = 3343)		LVEF of 45–54% (*n* = 1541)		LVEF of 35–44% (*n* = 966)		LVEF < 35% (*n* = 1038)		*p*-Value
**Age**, median (IQR)	68	(57–78) (*n* = 3343)	71	(59–79) (*n* = 1541)	72	(63–80) (*n* = 966)	70	(61–79) (*n* = 1038)	**0.001**
**Male sex**, *n* (%)	1964	(58.7)	1063	(69.0)	674	(69.8)	785	(75.6)	**0.001**
**Body mass index,** kg/m^2^, median (IQR)	27.6	(24.6–31.1) (*n* = 3343)	27.8	(24.8–31.3) (*n* = 1541)	26.8	(23.9–30.8) (*n* = 966)	26.7	(24.1–30.4) (*n* = 1038)	**0.001**
**Cardiovascular risk factors**, *n* (%)									
Arterial hypertension	2882	(86.2)	1428	(92.7)	879	(91.0)	861	(82.9)	**0.001**
Diabetes mellitus	793	(23.7)	422	(27.4)	287	(29.7)	278	(26.8)	**0.001**
Hyperlipidemia	1286	(38.5)	613	(39.8)	316	(32.7)	318	(30.6)	**0.001**
**Prior medical history**, *n* (%)									
Congestive heart failure	167	(5.0)	131	(8.5)	126	(13.0)	208	(20.0)	**0.001**
Pacemaker	8	(0.2)	17	(1.1)	23	(2.4)	52	(5.0)	**0.001**
COPD	125	(3.7)	57	(3.7)	43	(4.5)	43	(4.1)	0.718
Chronic kidney disease	142	(4.2)	87	(5.6)	66	(6.8)	88	(8.5)	**0.001**
Liver cirrhosis	39	(1.2)	10	(0.6)	13	(1.3)	10	(1.0)	0.293
Malignancy	162	(4.8)	85	(5.5)	81	(8.4)	54	(5.2)	**0.001**
Stroke	31	(0.9)	15	(1.0)	6	(0.6)	4	(0.4)	0.281
**Comorbidities at index hospitalization**, *n* (%)									
Acute coronary syndrome									
Unstable angina	1210	(36.2)	370	(24.0)	140	(14.5)	141	(13.6)	**0.001**
STEMI	210	(6.3)	271	(17.6)	206	(21.3)	144	(13.9)	**0.001**
NSTEMI	564	(16.9)	339	(22.0)	217	(22.5)	160	(15.4)	**0.001**
Atrial fibrillation	764	(22.9)	419	(27.2)	308	(31.9)	359	(34.6)	**0.001**
Atrial flutter	63	(1.9)	30	(1.9)	24	(2.5)	30	(2.9)	0.198
Acute decompensated heart failure	207	(6.2)	154	(10.0)	210	(21.7)	720	(69.5)	**0.001**
Cardiogenic shock	18	(0.5)	24	(1.6)	32	(3.3)	152	(14.6)	**0.001**
Atrioventricular block	85	(2.5)	37	(2.4)	28	(2.9)	22	(2.1)	0.721
Cardiopulmonary resuscitation	83	(2.5)	64	(4.2)	76	(7.9)	173	(16.7)	**0.001**
Out of hospital	62	(1.9)	43	(2.8)	55	(5.7)	112	(10.8)	**0.001**
In hospital	21	(0.6)	21	(1.4)	21	(2.2)	61	(5.9)	**0.001**
Valvular heart disease	498	(14.9)	258	(16.7)	213	(22.0)	301	(29.0)	**0.001**
Stroke	98	(2.9)	64	(4.2)	38	(3.9)	57	(5.5)	**0.001**

COPD, chronic obstructive pulmonary disease; LVEF, left ventricular ejection fraction; IQR, interquartile range; and (N)STEMI, non-ST-segment elevation myocardial infarction. Level of significance: *p* ≤ 0.05. Bold type indicates statistical significance.

**Table 2:** Procedural, laboratory and follow-up data.

	LVEF ≥ 55% (*n* = 3343)		LVEF of 45–54% (*n* = 1541)		LVEF of 35–44% (*n* = 966)		LVEF < 35% (*n* = 1038)		*p*-Value
**Coronary angiography**, *n* (%)									
No evidence of coronary artery disease	1331	(39.8)	359	(23.3)	196	(20.3)	251	(24.2)	**0.001**
One-vessel disease	703	(21.0)	312	(20.2)	199	(20.6)	167	(16.1)	
Two-vessel disease	617	(18.5)	353	(22.9)	201	(20.8)	219	(21.1)	
Three-vessel disease	692	(20.7)	517	(33.5)	370	(39.3)	401	(38.6)	
CABG	32	(1.0)	46	(3.0)	41	(4.2)	78	(7.5)	**0.001**
Chronic total occlusion	147	(4.4)	138	(9.0)	125	(12.9)	133	(12.8)	**0.001**
**Diseased vessels**, *n* (%)									
Right coronary artery	1250	(37.4)	825	(53.5)	527	(54.6)	570	(54.9)	**0.001**
Left main trunk	246	(7.4)	168	(10.9)	153	(15.8)	171	(16.5)	**0.001**
Left anterior descending	1498	(44.8)	907	(58.9)	640	(66.3)	653	(62.9)	**0.001**
Left circumflex	1092	(32.7)	745	(48.3)	475	(49.2)	524	(50.5)	**0.001**
Ramus intermedius	266	(8.0)	194	(12.6)	129	(13.4)	159	(15.3)	**0.001**
**PCI**, *n* (%)	1223	(36.6)	801	(52.0)	464	(48.0)	442	(42.6)	**0.001**
Right coronary artery	484	(14.5)	360	(23.4)	162	(16.8)	143	(13.8)	**0.001**
Left main trunk	86	(2.6)	49	(3.2)	52	(5.4)	58	(5.6)	**0.001**
Left anterior descending	624	(18.7)	376	(24.4)	268	(27.7)	240	(23.1)	**0.001**
Left circumflex	394	(11.8)	290	(18.8)	152	(15.7)	139	(13.4)	**0.001**
Ramus intermedius	48	(1.4)	28	(1.8)	19	(2.0)	23	(2.2)	0.314
CABG	8	(0.2)	12	(0.8)	7	(0.7)	22	(2.1)	**0.001**
**Sent to CABG**, *n* (%)	121	(3.6)	73	(4.7)	69	(7.1)	53	(5.1)	**0.001**
**Procedural data**									
Number of stents, *n* (%)	2	(1–3) (*n* = 1210)	2	(1–3) (*n* = 794)	2	(1–4) (*n* = 454)	2	(1–4) (*n* = 437)	**0.001**
Stent length, mm, median (IQR)	40	(23–68) (*n* = 1073)	44	(24–79) (*n* = 710)	50	(28–83) (*n* = 398)	48	(24–86) (*n* = 362)	**0.001**
**Baseline laboratory values**, median (IQR)									
Sodium, mmol/L	139	(138–141) (*n* = 3315)	139	(138–141) (*n* = 1532)	139	(138–141) (*n* = 960)	139	(137–141) (*n* = 1030)	0.379
Potassium, mmol/L	3.90	(3.70–4.13) (*n* = 3286)	3.95	(3.74–4.18) (*n* = 1525)	3.97	(3.75–4.21) (*n* = 956)	4.03	(3.78–4.30) (*n* = 1022)	**0.001**
Creatinine, mg/dL	0.960	(0.815–1.160) (*n* = 3318)	1.020	(0.865–1.251) (*n* = 1534)	1.11	(0.90–1.46) (*n* = 960)	1.253	(0.990–1.800) (*n* = 1030)	**0.001**
eGFR, mL/min/1.73 m^2^	72.64	(57.18–87.11) (*n* = 3318)	69.71	(52.10–84.52) (*n* = 1534)	63.83	(45.27–80.74) (*n* = 960)	57.35	(39.74–76.20) (*n* = 1030)	**0.001**
Urea, mg/dL	34.50	(27.80–44.75) (*n* = 3279)	36.78	(29.46–49.34) (*n* = 1520)	43.15	(31.78–62.15) (*n* = 957)	49.75	(36.96–74.41) (*n* = 1026)	**0.001**
Hemoglobin, g/dL	13.45	(12.10–14.60) (*n* = 3322)	13.30	(11.70–14.55) (*n* = 1532)	12.78	(11.10–14.20) (*n* = 964)	12.96	(11.10–14.40) (*n* = 1033)	**0.001**
WBC count, ×10^9^/L	8.41	(6.87–10.45) (*n* = 3322)	8.93	(7.20–11.15) (*n* = 1532)	9.57	(7.36–12.12) (*n* = 964)	9.76	(7.75–12.86) (*n* = 1033)	**0.001**
Platelet count, ×10^9^/L	238	(196–286) (*n* = 3321)	234	(191–279) (*n* = 1532)	234	(192–287) (*n* = 964)	228	(182–285) (*n* = 1033)	**0.001**
HbA1c, %	5.8	(5.4–6.4) (*n* = 1644)	5.8	(5.4–6.6) (*n* = 881)	5.9	(5.5–6.9) (*n* = 524)	6.0	(5.5–6.9) (*n* = 566)	**0.001**
LDL-cholesterol, mg/dL	110	(83–140) (*n* = 2271)	109	(81–140) (*n* = 1118)	98	(74–126) (*n* = 678)	94	(71–123) (*n* = 712)	**0.001**
C-reactive protein, mg/L	15.35	(6.70–53.55) (*n* = 2012)	24.15	(9.50–70.00) (*n* = 1117)	34.72	(11.23–96.25) (*n* = 792)	45.25	(14.30–109.10) (*n* = 863)	**0.001**
Albumin, g/L	35.40	(32.48–38.00) (*n* = 3265)	34.45	(31.21–37.05) (*n* = 1516)	33.00	(29.35–36.15) (*n* = 954)	31.70	(27.50–35.15) (*n* = 1024)	**0.001**
INR	1.04	(0.99–1.10) (*n* = 3264)	1.05	(1.01–1.12) (*n* = 1509)	1.08	(1.02–1.18) (*n* = 940)	1.13	(1.05–1.31) (*n* = 1012)	**0.001**
NT-pro BNP, pg/mL	670	(193–2214) (*n* = 1003)	1623	(499–3635) (*n* = 586)	3410	(1602–8554) (*n* = 446)	5208	(2375–11,869) (*n* = 603)	**0.001**
**All-cause mortality, in-hospital**	31	(0.9)	38	(2.5)	46	(4.8)	172	(16.6)	**0.001**
**Patients discharged alive**	3312	(99.1)	1503	(97.5)	920	(95.2)	866	(83.4)	
**Medication at discharge**, *n* (%)									
ACE inhibitor	1542	(46.6)	844	(56.3)	542	(59.2)	494	(57.5)	**0.001**
ARB	889	(26.9)	384	(25.6)	195	(21.3)	123	(14.3)	**0.001**
Beta-blocker	2049	(61.9)	1162	(77.5)	774	(84.6)	755	(87.9)	**0.001**
Aldosterone antagonist	149	(4.5)	113	(7.5)	266	(29.1)	502	(58.4)	**0.001**
ARNI	1	(0.0)	4	(0.3)	11	(1.2)	59	(6.9)	**0.001**
SGLT2 inhibitor	91	(2.8)	71	(4.7)	76	(8.3)	88	(10.2)	**0.001**
Statin	2385	(72.1)	1189	(79.3)	711	(77.7)	628	(73.1)	**0.001**
ASA	2096	(63.4)	1041	(69.4)	613	(67.0)	500	(58.2)	**0.001**
P2Y12 inhibitor	1376	(41.6)	866	(57.8)	510	(55.7)	374	(43.5)	**0.001**
OAK	784	(23.7)	436	(29.1)	304	(33.2)	345	(40.2)	**0.001**
**Follow-up data**, median (IQR)									
Hospitalization time	6	(4–11) (*n* = 3343)	7	(4–11) (*n* = 1541)	9	(5–15) (*n* = 966)	11	(6–19) (*n* = 1038)	**0.001**
ICU time	0	(0-0) (*n* = 3343)	0	(0-0) (*n* = 1541)	0	(0.0) (*n* = 966)	0	(0-0) (*n* = 1038)	**0.001**
**Primary endpoint**, *n* (%)									
Heart failure, at 36 months	397	(12.0)	326	(21.7)	342	(37.4)	417	(48.5)	**0.001**
**Secondary endpoints**, *n* (%)									
Acute myocardial infarction, at 36 months	141	(4.3)	111	(7.4)	89	(8.7)	184	(21.4)	**0.001**
Coronary revascularization, at 36 months	251	(7.6)	152	(10.1)	91	(9.9)	66	(7.7)	**0.008**

ACE, angiotensin-converting enzyme; ARB, angiotensin receptor blocker; ARNI, angiotensin receptor neprilysin inhibitor; ASA, acetylsalicylic acid; CABG, coronary artery bypass grafting; eGFR, estimated glomerular filtration rate; HbA1c, glycated hemoglobin; ICU, intensive care unit; IQR, interquartile range; LDL, low-density lipoprotein; NT-pro BNP, aminoterminal pro-B-type natriuretic peptide; PCI, percutaneous coronary intervention; SGLT2, sodium–glucose linked transporter 2; and WBC, white blood cell. Level of significance: *p* ≤ 0.05. Bold type indicates statistical significance.

**Table 3:** Multivariable Cox regression analyses with regard to risk of heart-failure-related rehospitalization, myocardial infarction, and coronary revascularization at 36 months.

	HF-Related Rehospitalization			Coronary Revascularization			AMI		
Variables	HR	95% CI	*p*-Value	HR	95% CI	*p*-Value	HR	95% CI	*p*-Value
Age (per year increase)	1.012	1.008–1.017	**0.001**	1.014	0.903–1.139	0.808	0.990	0.983–0.997	**0.005**
Male sex	1.020	0.887–1.173	0.778	1.025	0.906–1.161	0.695	0.923	0.764–1.116	0.408
Diabetes mellitus	1.151	1.004–1.319	**0.044**	1.332	1.186–1.496	**0.001**	1.234	1.031–1.477	**0.022**
Prior coronary artery disease	1.631	1.350–1.971	**0.001**	1.175	0.883–1.564	0.268	1.215	0.919–1.606	0.171
Prior myocardial infarction	0.989	0.672–1.456	0.956	1.622	1.250–2.105	**0.001**	1.648	1.043–2.605	**0.032**
Prior CABG	1.278	1.022–1.597	0.032	1.234	0.882–1.728	0.220	1.248	0.923–1.688	0.150
Chronic kidney disease	1.319	1.066–1.631	**0.011**	1.588	1.328–1.899	**0.001**	0.959	0.676–1.361	0.816
STEMI	0.924	0.693–1.232	0.588	0.803	0.545–1.182	0.266	0.798	0.621–1.027	0.079
NSTEMI	0.945	0.796–1.123	0.520	0.959	0.758–1.213	0.727	0.751	0.595–0.948	**0.016**
Atrial fibrillation	1.223	1.066–1.403	**0.004**	1.185	1.055–1.331	**0.004**	0.974	0.796–1.193	0.801
Decompensated heart failure	1.607	1.374–1.880	**0.001**	1.490	1.287–1.725	**0.001**	1.451	1.161–1.812	**0.001**
LVEF of 54–45%	1.826	1.573-2.121	**0.001**	1.172	0.952-1.443	0.136	1.667	1.292-2.150	**0.001**
LVEF of 44–35%	2.948	2.523-3.446	**0.001**	1.084	0.836-1.405	0.543	1.988	1.497-2.640	**0.001**
LVEF < 35%	3.731	3.168-4.394	**0.001**	0.867	0.632-1.190	0.378	4.184	3.200-5.471	**0.001**
LVEF ≥ 55%	(Reference group)			(Reference group)			(Reference group)		

CABG, coronary artery bypass grafting; CI, confidence interval; HR, hazard ratio; HF, heart failure; AMI, acute myocardial infarction; and LVEF, left ventricular ejection fraction. Level of significance: *p* ≤ 0.05. Bold type indicates statistical significance.

**Table 4:** Multivariable Cox regression analyses with regard to the risk of heart-failure-related rehospitalization at 36 months in pre-specified subgroup.

		Heart-Failure-Related Rehospitalization		
Variable		HR	95% CI	*p*-Value
Age ≥ 70 years	LVEF < 35%	2.338	1.793–3.050	**0.001**
	LVEF of 44–35%	2.007	1.558–2.586	**0.001**
	LVEF of 54–45%	1.512	1.182–1.935	**0.001**
	LVEF ≥ 55%	(Reference group)		
Age < 70 years	LVEF < 35%	3.874	2.783–5.393	**0.001**
	LVEF of 44–35%	2.925	2.043–4.189	**0.001**
	LVEF of 54–45%	1.802	1.256–2.585	**0.002**
	LVEF ≥ 55%	(Reference group)		
Male sex	LVEF < 35%	3.363	2.459–4.599	**0.001**
	LVEF of 44–35%	2.599	1.900–3.555	**0.001**
	LVEF of 54–45%	1.786	1.317–2.422	**0.001**
	LVEF ≥ 55%	(Reference group)		
Female sex	LVEF < 35%	3.061	2.048–4.579	**0.001**
	LVEF of 44–35%	2.217	1.515–3.246	**0.001**
	LVEF of 54–45%	1.455	1.021–2.074	**0.038**
	LVEF ≥ 55%	(Reference group)		
Unstable angina	LVEF < 35%	4.214	2.800–6.340	**0.001**
	LVEF of 44–35%	3.531	2.247–5.550	**0.001**
	LVEF of 54–45%	1.979	1.234–3.174	**0.005**
	LVEF ≥ 55%	(Reference group)		
STEMI	LVEF < 35%	4.028	2.351–6.892	**0.001**
	LVEF of 44–35%	2.973	1.682–5.255	**0.001**
	LVEF of 54–45%	1.778	1.001–3.159	**0.050**
	LVEF ≥ 55%	(Reference group)		
NSTEMI	LVEF < 35%	3.420	2.349–4.979	**0.001**
	LVEF of 44–35%	2.346	1.572–3.501	**0.001**
	LVEF of 54–45%	1.624	1.123–2.348	**0.010**
	LVEF ≥ 55%	(Reference group)		
Decompensated heart failure	LVEF < 35%	5.612	3.950–7.972	**0.001**
	LVEF of 44–35%	3.694	2.525–5.402	**0.001**
	LVEF of 54–45%	1.938	1.291–2.910	**0.001**
	LVEF ≥ 55%	(Reference group)		
No/–one-vessel disease	LVEF < 35%	3.145	2.326–4.254	**0.001**
	LVEF of 44–35%	2.255	1.630–3.121	**0.001**
	LVEF of 54–45%	1.405	1.035–1.907	**0.029**
	LVEF ≥ 55%	(Reference group)		
Two/three-vessel disease	LVEF < 35%	4.847	3.049–7.708	**0.001**
	LVEF of 44–35%	3.277	2.047–5.243	**0.001**
	LVEF of 54–45%	1.720	1.041–2.841	**0.034**
	LVEF ≥ 55%	(Reference group)		

CI, confidence interval; HR, hazard ratio; LVEF, left ventricular ejection fraction; STEMI, ST-elevation myocardial infarction; and NSTEMI, non-ST-elevation myocardial Infarction. Level of significance: *p* ≤ 0.05. Bold type indicates statistical significance.

## Data Availability

The datasets used and/or analyzed during the current study are available from the corresponding author upon reasonable request.
